# Population-specific common SNPs reflect demographic histories and highlight regions of genomic plasticity with functional relevance

**DOI:** 10.1186/1471-2164-15-437

**Published:** 2014-06-06

**Authors:** Ananyo Choudhury, Scott Hazelhurst, Ayton Meintjes, Ovokeraye Achinike-Oduaran, Shaun Aron, Junaid Gamieldien, Mahjoubeh Jalali Sefid Dashti, Nicola Mulder, Nicki Tiffin, Michèle Ramsay

**Affiliations:** Sydney Brenner Institute of Molecular Bioscience, University of the Witwatersrand, Johannesburg, South Africa; Division of Human Genetics, National Health Laboratory Service, School of Pathology, Faculty of Health Sciences, University of the Witwatersrand, Johannesburg, South Africa; School of Electrical & Information Engineering, University of the Witwatersrand, Johannesburg, South Africa; Department Clinical Laboratory Sciences, Computational Biology Group, IDM, University of Cape Town, Cape Town, South Africa; South African National Bioinformatics Institute/Medical Research Council of South Africa Bioinformatics Unit, University of the Western Cape, Bellville, South Africa

## Abstract

**Background:**

Population differentiation is the result of demographic and evolutionary forces. Whole genome datasets from the 1000 Genomes Project (October 2012) provide an unbiased view of genetic variation across populations from Europe, Asia, Africa and the Americas. Common population-specific SNPs (MAF > 0.05) reflect a deep history and may have important consequences for health and wellbeing. Their interpretation is contextualised by currently available genome data.

**Results:**

The identification of common population-specific (CPS) variants (SNPs and SSV) is influenced by admixture and the sample size under investigation. Nine of the populations in the 1000 Genomes Project (2 African, 2 Asian (including a merged Chinese group) and 5 European) revealed that the African populations (LWK and YRI), followed by the Japanese (JPT) have the highest number of CPS SNPs, in concordance with their histories and given the populations studied. Using two methods, sliding 50-SNP and 5-kb windows, the CPS SNPs showed distinct clustering across large genome segments and little overlap of clusters between populations. iHS enrichment score and the population branch statistic (PBS) analyses suggest that selective sweeps are unlikely to account for the clustering and population specificity. Of interest is the association of clusters close to recombination hotspots. Functional analysis of genes associated with the CPS SNPs revealed over-representation of genes in pathways associated with neuronal development, including axonal guidance signalling and CREB signalling in neurones.

**Conclusions:**

Common population-specific SNPs are non-randomly distributed throughout the genome and are significantly associated with recombination hotspots. Since the variant alleles of most CPS SNPs are the derived allele, they likely arose in the specific population after a split from a common ancestor. Their proximity to genes involved in specific pathways, including neuronal development, suggests evolutionary plasticity of selected genomic regions. Contrary to expectation, selective sweeps did not play a large role in the persistence of population-specific variation. This suggests a stochastic process towards population-specific variation which reflects demographic histories and may have some interesting implications for health and susceptibility to disease.

**Electronic supplementary material:**

The online version of this article (doi:10.1186/1471-2164-15-437) contains supplementary material, which is available to authorized users.

## Background

The global diversity of human genomes is the outcome of a series of demographic and evolutionary events including migration, bottlenecks, admixture, population isolation, natural selection and genetic drift which occurred in different parts of the world at various time points in history [1–3]. Genomic signatures of many of these events have been preserved in the genomes of different populations and play a pivotal role in uncovering demographic histories in addition to understanding health and disease [4, 5]. In the last decade, two major large consortium based efforts; the HapMap project, the Human Genome Diversity Project (HGDP), as well as several other studies, based on genotyping of single nucleotide changes, have attempted to catalogue the genetic variations that exist between individuals of a population as well as within different populations across continents [6–11].

Data from these studies on genetic diversity have been instrumental in estimating the origin and history of different contemporary populations as well as shedding light on the evolutionary relationship between them [12]. Moreover, the genotype data from these studies have been subjected to various computational techniques to derive estimates of population sizes and divergence times for the major demographic events in human history, which in many cases have been found to be in agreement with evidence from existing historical accounts and archaeological records [13, 14]. However, these studies were based on a fixed number of single nucleotide polymorphisms (SNPs) which had clear ascertainment bias (the SNPs included in the genotyping platforms were selected on the basis of their occurrence and frequencies primarily in European populations), therefore it was difficult to reliably assess the nature and extent of genomic diversity that exists among different populations from these studies [15].

The next major wave of information about genetic and genomic diversity in human populations came from studies based on exome and whole genome sequencing [16–19]. The 1000 Genomes Project, for example, in addition to identifying millions of novel SNPs and more than a million short structural variants (SSVs), showed that rare variants account for a large majority of the existing genetic diversity between individuals as well as within populations [17, 18]. Moreover, it was suggested that there is an excess of rare and deleterious mutations in human genomes, probably resulting from exponential population growth and weak purifying selection [17, 18]. Studies based on deep sequencing of selected regions from thousands of individuals further show that the majority of rare coding variants, with allele frequencies lower than 0.0005, are also population-specific and potentially deleterious [19]. In addition to thousands of contemporary human genomes, sequencing of many archaic genomes has also been performed recently which has provided evidence for archaic admixture in non-African genomes [20–22]. Such admixture might also be present in at least some of the African populations [23, 24]. These studies taken together have not only resulted in a paradigm shift in our understanding of various aspects of human genomic diversity but also provided necessary data for addressing numerous other questions related to human genome evolution.

SNPs and structural variants are broadly classified into common and rare based on minor allele frequencies (MAF). A widely used cut-off for defining rare SNPs being a MAF of less than 0.05 [17]. However, this cut-off is pragmatic in nature and does not have any special biological relevance. Although differences in SNP allele frequencies might be influenced by various demographic factors like selection and population size, time is the major determinant in the rise or fall of allele frequencies. Mathematical estimates suggest most of the common SNPs to have originated thousands of years ago and therefore to have a wider geographic distribution in contrast to rare variants which are mostly more recent and geographically restricted [25]. The rare and common variants therefore allow us to investigate events at different time scales of demographic histories. The relative phenotypic importance of common and rare SNPs is highly debated [26]. Nevertheless, while most of the Mendelian traits and deleterious mutations have been shown to be rare; several studies suggest some continuous traits like height might well be explained in terms of common SNPs [27, 28].

SNPs and structural variants are often classified into ‘private’ and ‘shared’ based on their distribution in a single population or a range of populations. The term private however might imply different things based on the context, for example, a SNP might be private to an individual or a family, or to a population (monomorphic in all but one population; also referred to as ‘population private’) or to an ancestral group. Therefore, we will use the term ‘population-specific’ for the SNPs that have been found to occur only in a single population. Although private SNPs have not been shown to be involved in major phenotypic traits or common diseases, population-specific SNPs might well be important in ascribing characteristic phenotypes and disease susceptibility/protection to a population [29, 30].

Population specificity of genetic variants, if the population-specific allele is the derived allele, might originate from two different scenarios: in the first scenario, a variant allele originates in a single population and remains restricted to the population of its origin. The second scenario is that the variant originated before differentiation of populations, survives in only a single population, and gets eliminated from other populations. In cases where the population-specific allele is the ancestral allele, both the alleles are estimated to have evolved far back in evolutionary history and the derived allele replaces the ancestral allele in all but one of the populations, probably through selective sweeps. Alternatively, in some cases, the assignment of ancestral state may be incorrect. The other possible scenario by which population-specific SNPs might originate is by admixture with populations which are not included in the study or even populations which are no longer extant. Therefore, in addition to the functional role of these SNPs, the population-specific SNPs might also play an important role in characterizing ancestry and understanding demographic histories [31, 32]. For example, on a genome wide scale the number of population-specific SNPs in a population would be expected to be related to the age of the population and also to reflect demographic events like bottlenecks, geographical isolation and admixtures.

Despite their potential significance, population-specific SNPs have not been studied extensively. Previous HapMap data based studies on population-specific SNPs have been able to identify only a small number of population-specific SNPs due to ascertainment bias of the genotyping platform [6, 33–35]. The availability of unbiased whole genome sequence data from sources like the 1000 Genomes project, however, has now made the identification and characterization of population-specific SNPs on a genome wide scale possible. Moreover, sequencing-based studies have shown population-specific SNPs to be one of the major components of genetic diversity within populations [17–19, 36]. A deeper understanding of population-specific variations, their genomic distribution and potential functional relevance is important.

We have used 1000 Genomes sequence data (release October 2012), including more than one thousand individuals from 14 populations spanning Europe, Asia, Africa and America, to identify SNPs and structural variants that are private or specific to each population and to study their genomic distribution and potential functional relevance [17, 18]. However, as the population sample sizes are relatively small (<100) and the sequencing is low coverage (4X-6X) for most of the 1000 Genomes data, low frequency alleles are harder to accurately identify and may be incorrectly identified as population-specific [17, 18]. We have therefore focused our study on common population-specific (CPS) SNPs as higher MAF population-specific SNPs are expected to be more informative and less likely to be incorrectly annotated as population-specific in this dataset. We evaluated the frequency distribution of population-specific SNPs identified in our study in the context of the generally accepted model of population migration and differentiation. We analysed the genomic distribution of these SNPs using fixed length and fixed bin window scan based approaches to identify potential biases in genomic distribution of CPS SNPs. The CPS SNP-enriched genomic regions in different populations were then compared to test whether their preferential localization has overlaps across different populations. Analyses of signatures of selection and the distribution of recombination hotspots were performed in the CPS SNP-enriched genomic regions to determine the extent of involvement of these processes in generating CPS SNP-enriched genomic regions in different populations. Functional enrichment analysis of genes containing the CPS SNP was performed and the enriched functional classes for different populations were compared to identify possible functional trajectories in population-specific SNP evolution.

## Results and discussion

### Identifying SNPs unique to each population

One of the major achievements of the 1000 Genomes project has been the identification of numerous novel SNPs across different populations [17, 18]. The sequence-based approach employed in the 1000 Genomes project in contrast to the previous genotyping-based approaches like HGDP and HapMap, provides an unbiased estimate of human genetic variation across many populations globally [6, 7, 17, 18]. We have used the most recent version (October 2012) of the 1000 Genomes data to identify SNPs which are observed to be unique to each of the individual study populations [18]. These SNPs were categorized into CPS SNPs and rare population-specific (RPS) SNPs based on a MAF cut-off of 0.05. SNPs with MAF >0.05 were considered as CPS SNPs while SNPs with lower MAFs were considered as RPS SNPs. Although more than 99% of population specific SNPs in the 1000 Genomes data are RPS SNPs, we have focused our present study on CPS SNPs because the sample sizes (around 90–100 individuals for each population) and low coverage sequencing (around 4X for most of the genomic regions) used for generating the data make it difficult to reliably ascertain the population specificity of low allele frequency SNPs. Moreover, as these SNPs have a MAF of at least 0.05 they are less likely to be personal SNPs or the result of recent demographic events.

**Figure 1:**
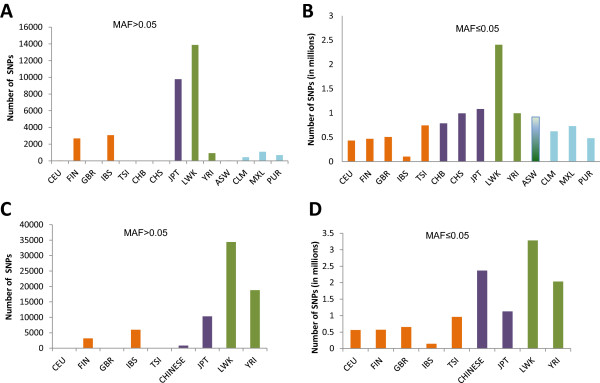
**Population-specific SNPs in the 1000 genomes data.** The number of population-specific SNPs for each of the 14 populations for common **(A)** and rare **(B)** SNPs are shown in **(A)** and **(B)**. As the dataset includes admixed and related populations we removed the four known admixed populations (ASW, CLM, PUR, and MXL) and merged the two Chinese populations CHS and CHB into a single CHINESE population. The number of common **(C)** and rare **(D)** population-specific SNPs in the remaining 9 populations were retained for further analysis. The European populations are shown in orange, Asian populations in purple and the African populations in light green. The American and the admixed African populations are shown in blue.

The present 1000 Genomes data contain two African (YRI (Yoruba in Ibadan, Nigeria), LWK (Luhya in Webuye, Kenya)), three Asian (JPT (Japanese in Tokyo, Japan), CHB (Han Chinese in Beijing, China) and CHS (Han Chinese South)), three American (MXL (Mexican Ancestry in Los Angeles, CA, USA), PUR (Puerto Ricans in Puerto Rico) and CLM (Colombians in Medellín, Colombia)), 5 European (IBS (Iberian Populations in Spain), GBR (British from England and Scotland), CEU (Utah residents with ancestry from northern and western Europe), FIN (Finnish in Finland) and TSI (Toscani in Italia)) and one admixed African (ASW (African Ancestry in SW USA)) population. The frequencies of common and rare population-specific SNPs in these populations have been summarized in Figure 1A and Figure 1B**,** respectively. Although the numbers of common and rare SNP differ by many folds, there are some broad similarities in the distribution patterns of the CPS SNPs and RPS SNPs.

For example, the highest number for both CPS SNPs and the RPS SNPs was observed in the LWK population followed by the Japanese (JPT) population. Interestingly, in contrast to the large number of RPS SNPs observed, just a few CPS SNPs were found to occur in the Chinese populations (CHB and CHS). This observation is consistent with the fact that these populations have a similar geographic origin, and the differentiation between them probably started little more than a thousand years ago with the Southward migration of the Northern Han population [37–39]. In spite of the pronounced divergence of these populations, reflected in the high frequency of RPS SNPs and has also been observed in many previous studies, the relatively recent divergence has not allowed many of the population-specific alleles to reach frequencies of 0.05 [37–39]. As our aim was to identify common SNPs which are unique in different populations, and we know that these populations have a common recent origin, we merged the two Chinese populations CHB and CHS into a single population (named CHINESE for this study). We recognise that this approach would not be suitable for a similar analysis with rare SNPs due to the extent of divergence that these populations have undergone recently.

One of the concerns with using all the current populations of the 1000 Genomes data for identifying population-specific SNPs is the inclusion of populations with known recent admixture, such as ASW and MXL (Supplementary Figures S4 and S9 from reference 18). The inclusion of these admixed populations may mask the true population specificity of SNPs. In order to identify SNPs which are truly unique to populations, ASW and the three American populations (MXL, PML and PUR), which are known to have undergone a significant amount of admixture in the recent past, were removed from the dataset [18]. It is worth noting, however, that the MXL, CLM and PUR populations contain a few hundred common SNPs which were not observed in any other continent/population. As indicated by previous population structure analyses, these populations harbour a significant Native American genetic component; and the order of Native American admixture in these three populations is approximated by the total number of population-specific SNPs in these populations (highest in MXL followed by CLM and then PUR) [18]. It would be an interesting follow-up study to isolate the population-specific SNPs of Native American origin and to functionally assess their significance in these populations.

The trimming and rearrangement of the population datasets resulted in 9 potentially independent and essentially non-admixed populations for further investigation in the current study. The distribution of the CPS SNPs and RPS SNPs for each population was recalculated considering these 9 populations only, and has been summarized in Figure 1C and D. The list of SNPs which were observed to be unique to each population along with their frequencies in the 14 study populations has been provided in Additional file 1. Although the removal of the admixed populations significantly increased the count of CPS SNPs for all the populations, the detected trends, for example the highest number of SNPs in LWK, followed by JPT, IBS and FIN, are similar in both sets (Figure 1A, C, B and D). An interesting exception is the YRI population, where the number of YRI specific CPS SNPs goes up by folds with the removal of the admixed African American population. This result concurs with the known history of recent migration and admixture of the Western African populations in North America [40]. However, in spite of this increase in the number of the CPS SNPs in the YRI, after removal of admixed populations, they still have only about half the number of CPS SNPs observed in LWK. This difference is, however, not surprising in view of the fact that a number of different populations, which most probably include the LWK along with other Bantu-speaking populations, have migrated to East Africa at different time points in history [41–44]. The migration of several different populations along with the presence of indigenous East-African Khoesan-speaking populations in this region, which has been suggested to have contributed to the population differentiation in East-Africa, might also explain the high frequency of CPS SNPs and RPS SNPs observed in the LWK [41, 42].

The relatively high frequency of CPS SNPs as well as RPS SNPs in the Japanese population is notable. It is well known that the modern Japanese population contains admixtures of at least two distinct genetic components; the old migrants who migrated to the Japanese Archipelago approximately 30,000 years ago and the new migrants that reached Japan only about a couple of thousand years ago [45–47]. It would be interesting to study how far the unique components of both these, and perhaps other migrating populations, add up to generate the high RPS SNPs and CPS SNPs observed in the JPT population.In addition to population histories, the sample size is also a strong determinant of how many variants and unique variants are observed in a population. For example, the huge increase in the frequency of RPS SNPs in the Chinese populations after the merger (Figure 1D) is also an outcome of the increase in sample size due to merging of the populations. As the sample size for the population has doubled the frequency of detection of RPS SNPs has increased proportionately and similar changes can be expected to be observed in other populations in the future as more samples from these populations are sequenced. Similarly, the lack of RPS SNPs in the IBS population in comparison to other populations can be ascribed to the inclusion of only 14 IBS samples in the current 1000 Genomes data set. It can be expected that as more samples are sequenced the fraction of RPS SNPs in this population will be in line with other populations.

**Figure 2:**
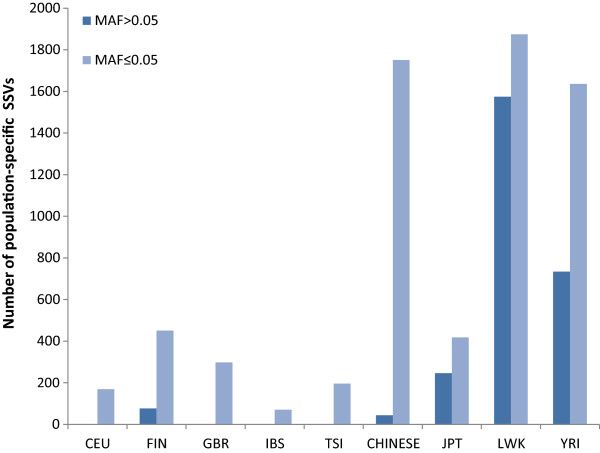
**Population-specific common (MAF > 0.05) and rare (MAF ≤ 0.05) short structural variants (SSVs).**

We found that three of the European populations (CEU, GBR and TSI) have only a handful of common SNPs unique to them in contrast to a few hundred thousand rare SNPs. While this makes sense in terms of demographics [48, 49] and probable admixtures, it might also be a result of treating these related or partially admixed populations separately. Approaches that group these populations together, based on population histories, might lead to the identification of some CPS SNPs in these groups too. While the high frequency of CPS SNPs in the Finnish population (FIN) can be interpreted in terms of multiple genetic components and demographic factors like isolation, migration and admixture, which is reflected in their distinctive distribution in the European principal component analysis (PCA) plots in other studies [18, 50, 51], the high frequency of CPS SNPs in the Spanish (IBS) population needs to be treated with greater caution as the number of individuals sequenced for this population is only 14. Many of the SNPs which seem to be common (MAF > 0.05) in the IBS in the present data might turn out to be rare once other samples from this population are sequenced.Although our analysis is focused on SNPs, we studied the distribution of population-specific short structural variants (SSVs) to see whether their distribution in different populations concurs with that of the SNPs. Figure 2 shows the distribution of the common population specific structural variants (CPS SSVs) and rare population-specific structural variants (RPS SSVs). Interestingly, the relative prevalence of the SSVs across populations shows high concordance with that of SNPs. However, the numbers observed for rare and common SSVs are similar in contrast to the few fold difference observed in the number of common and rare SNPs.

**Figure 3:**
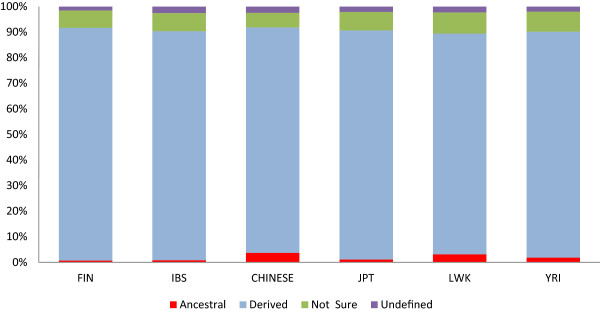
**Classification of population-specific SNP alleles into ancestral and derived.** The SNPs for which no ancestral state information could be detected are shown as “Undefined” whereas the SNPs for which the ancestral state could not be detected with confidence are shown as “Not Sure”.

To classify the CPS SNP variant alleles into ancestral and derived (based on multi-species alignment) the ancestral/derived information for alleles in the 1000 Genomes vcf file was used [18]. As expected, more than 80% of the population-specific alleles were found to be the derived allele (Figure 3) indicating that most of these alleles likely arose in the individual populations after their divergence from other populations.

The relative prevalence of the CPS SNPs (as well as RPS SNPs and SSVs) across populations, therefore, shows high concordance with what can be expected on the basis of the generally accepted model of population divergence and the relationships between populations. However, as has been demonstrated, the number of population-specific SNPs observed in any population, in addition to population histories, is also influenced by factors like sample size and number of related and/or admixed populations included in the study. The removal of the admixed African and American populations almost doubled the number of common SNPs which were detected to be population-specific in the other 9 populations, indicating how important the detection and control of admixture is for identifying what is truly population-specific. While the lack of CPS SNPs in most European populations is not very surprising considering their population histories, as well as the number of populations (5 European populations in contrast to only 2 African and 3 Asian population) included in the dataset, it would be interesting to see how strongly the inclusion of other populations from Asia and Africa change the number of population-specific SNPs as new data pour in.

### Genomic distribution of CPS SNPs

The distribution of SNPs has for long been known to be non-random across the genome [52–55]. Recent studies have further suggested that the rates of mutations in a genomic region in addition to the genomic context might also depend on the presence of repeat sequences and even existing SNPs in the region [56, 57]. Moreover, genomic regions where genomes from different global populations differ very strongly from each other have also been observed [58]. Given this background it was interesting to investigate whether the CPS SNPs, as delineated in our study, also show clustered occurrences across the genome. To identify possible biases in the distribution of CPS SNPs in each population and test whether the enriched regions are similar in different populations we used a sliding window based scan. Although sliding window based approaches have been widely used to identify clusters within genomic regions [59, 60], this approach has been shown to find some false positive clusters in some cases [61]. Therefore, to minimize such false positive results we have used two different sliding windows based approaches and used a conservative *p*-value cut-off for delineating clusters of CPS SNPs in each population.

#### 50-SNP windows

In the first approach, a window was defined as a set of 50 contiguous SNPs and each chromosome was scanned along the 50-SNP windows (with a slide of 50 SNPs per step) separately for each population. In each step the fraction of CPS SNPs in each window was recorded and compared to an expected value, based on the occurrence of CPS SNPs on the corresponding chromosome for the particular population. The statistical significance of the observations was estimated using cumulative hyper geometric *p*-values calculated for each window. The results clearly identified specific regions of the genome to be enriched with CPS SNPs in each population. We detected 655 CPS SNP-enriched windows/regions in the 6 populations (Table 1, Additional file 2). The populations CEU, TSI and GBR were not analysed due to a paucity of CPS SNPs. As for the number of CPS SNPs in the population, most CPS SNP-enriched windows were observed in the LWK, followed by YRI and JPT. It is interesting to note that, although both FIN and IBS contain a much greater number of CPS SNPs in comparison to the CHINESE population, which contains 24 enriched windows, only three CPS SNP-enriched windows were detected in the IBS population and a single such window was detected in the FIN population. The two highest-scoring windows detected for each population using this scan are shown in Table 2. In the highest-scoring windows for both LWK and YRI more than 50% of the SNPs were found to be CPS SNPs.

**Table 1:** **Genomic regions enriched in common population-specific (CPS) SNPs identified using 50-SNP and 5-kb window approaches**

Population	Sample size	CPS SNPs	50-SNP window	5-Kb window	Overlap
LWK	97	34390	357	311	237
YRI	88	18809	216	188	138
JPT	89	10326	64	47	41
CHINESE	197	863	24	28	21
FIN	93	3178	1	1	0
IBS	14	5971	3	1	0
Total		73537	665	576	437

The populations CEU, TSI and GBR were excluded from this analysis due to low numbers of CPS SNPs in these populations.

**Table 2:** **Best common population-specific (CPS) SNP-enriched windows for each population**

Population	Chr	Start	End	No. of SNPs	P-value	Gene or flanking genes
YRI	18	6266587	6271281	26	4.36E-66	*L3MBTL4*
YRI	12	21347746	21353031	25	9.95E-60	*SLCO1B1*
LWK	10	26690276	26697294	26	1.19E-59	*GAD2 - APBB1IP*
LWK	3	132399187	132404788	25	3.74E-56	*NPHP3-ACAD11*
JPT	2	38809530	38818371	20	5.77E-46	*HNRPLL*
JPT	4	187416152	187426671	18	7.30E-44	*LOC285441*-*MTNR1A*
CHINESE	2	152284636	152297774	12	8.13E-36	*RIF1*
CHINESE	11	119411414	119420288	11	2.13E-33	*LOC100499227*- *PVRL1*
FIN	16	86084552	86093750	4	2.45E-09	*IRF8*-*LOC146513*
IBS	3	68079	77942	5	6.40E-10	na-*CHL1*
IBS	19	52867878	52878655	4	2.77E-08	*ZNF610*

Population code, genomic coordinates, number of CPS SNPs, *p*-values and corresponding genes (if window is exonic or intronic) or flanking genes joined by a “-“ (if the window is intergenic), for up to two best 50-SNP windows for each population.

Intergenic window for which no flanking gene was found is indicated by “na”.

#### 5-kb windows

**Figure 4:**
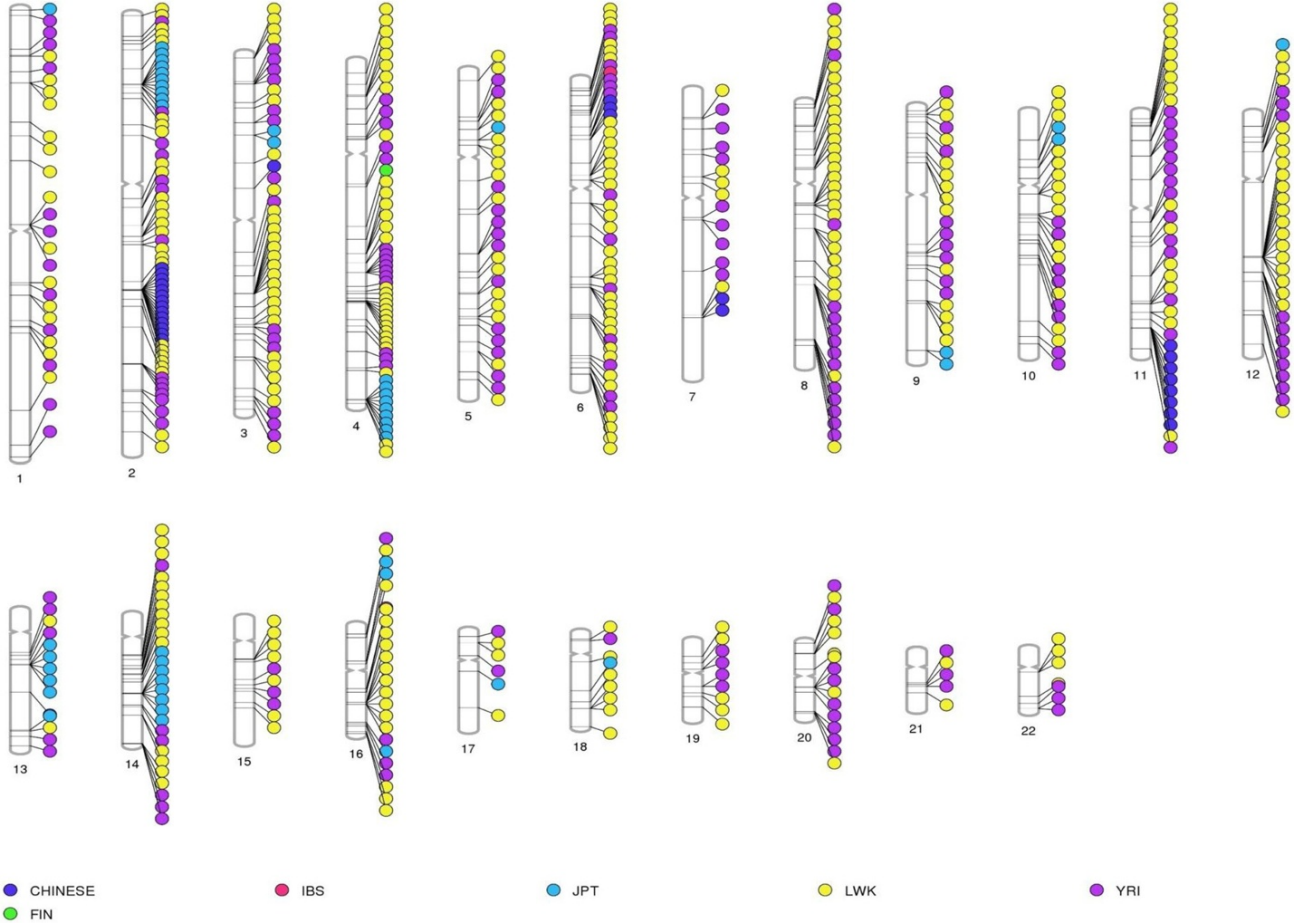
**Genomic distribution of common population-specific (CPS) SNP-enriched 5-kb windows.** The windows show very little overlap between populations and there are many blocks within populations containing contiguous windows of CPS SNP enrichment.

The second approach was to use a sliding window of 5 kilobases (kb). This approach, in addition to identifying CPS SNP-enriched regions, provides a more direct way to identify possible overlap within CPS SNP-enriched windows across populations. Using this scan, 565 5-kb regions were found to be significantly enriched for CPS SNPs in the 6 populations (Table 1). For each of the populations there was a very significant amount of overlap between the regions identified by the two sliding window based approaches (Table 1). The comparison of enriched windows identified using both the sliding window approaches shows that there is almost no overlap within the CPS SNP-enriched regions in these six populations (Figure 4). The second interesting aspect revealed by both the 50-SNP windows and 5-kb windows based approaches is that for many genomic regions the run of enrichment extends far beyond a single or couple of windows. The regions containing the longest stretches of enriched 50-SNP windows have been summarized in Table 3.

**Table 3:** **Longest CPS SNP-enriched 50-SNP window stretch for each population**

Population	Chromosome	Start	End	Block length	Gene or flanking genes
YRI	12	21343612	21361661	6	*SLC01B1*
JPT	4	187420496	187467709	11	*MTNR1A*
LWK	12	79979498	80083792	12	*PAWR*
CHINESE	2	152268276	152401521	14	*RIF1*

Population code, genomic coordinates; number of 50-SNP windows in the block and the related loci are shown for each population.

No such blocks were observed for the FIN and the IBS populations.

Interestingly, the longest blocks and the highest scoring windows show significant overlap in some populations (Tables 2 and 3). For example, one of the longest blocks as well as one of the most CPS SNP dense windows was detected near the solute carrier organic anion transporter family, member 1B1 (*SLCO1B1*) gene in the YRI population. Sequence variants identified in the *SLCO1B1* gene have been associated with altered transport activity and it has been shown that genetic polymorphisms in the gene have an impact on the inter-individual variability of the pharmacokinetics and pharmacodynamics of specific drugs [62, 63]. Previous studies have also observed unique genetic diversity in the *SLCO1B1* gene between populations with the greatest diversity among African populations [62, 63]. Similar overlap was also observed in the RAP1 interacting factor homolog (*RIF1*) gene in the CHINESE population**.** Additional files 2 and 3 contain the full list of windows identified using these approaches, and the SNPs included in them. Interestingly, despite fewer CPS SNPs and the presence of only a few enriched windows, two significantly long stretches of enrichment are observed in the CHINESE population. Similarly, although the number of enriched windows in Japanese is less than one third of that of the YRI, the Japanese population seem to harbour much longer enriched window stretches in comparison to the YRI population, and this enrichment cannot be explained solely on the basis of increased LD in the Japanese compared to the YRI These observations taken together indicate that the bias in distribution of CPS SNPs is largely independent of the size of the datasets and the enriched windows or window blocks may represent genomic regions significant in terms of function or population histories.

### Possible origin of CPS SNP-enriched genomic regions

Clusters of SNPs with highly differentiated allele frequencies, within and between species, have been observed in numerous previous studies [64–66]. The origin of such clusters has been ascribed to various demographic factors like genetic drift and gene flow as well as forces like selection and local adaptations [67–69]. The CPS SNP clusters observed in our study are somewhat similar to the clusters which show high allele frequency differentiation within populations as they represent genomic regions which vary widely across populations. However, there is an inherent difference in that in these regions both the SNP composition and SNP density is different in a single population compared to others. Considering this background it was important to investigate if the factors, which are assumed to generate clusters of SNPs with highly differentiated allele frequencies across populations, are also responsible for generating clusters of CPS SNPs. We used different computational approaches to test possible involvement of selection or increased recombination rates in the origin of these clusters.

#### Role of selective sweeps

**Figure 5:**
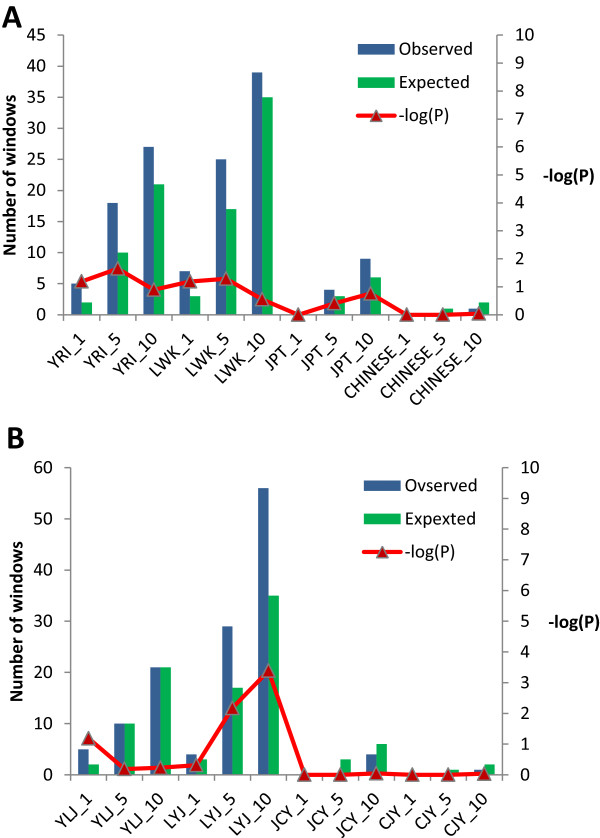
**Analysis of potential signatures of selection in the common population-specific (CPS) SNP-enriched windows. (A)** Expected and observed number of iES (iHS enrichment score) enriched windows (see Methods for details) in YRI, LWK, JPT and CHINESE populations. The number which has been appended to the population code indicates the top n^th^ percentage of iHS score considered (1 = top 1%; 5 = top 5% and 10 = top 10%). The corresponding *p*-values for enrichment are shown on the right axis. **(B)** Expected and observed occurrences of top 1%, 5% and 10% population branch statistic (PBS) scores amongst CPS SNP-enriched windows for YRI, LWK, JPT and CHINESE populations. A three letter population combination code (say YLJ) has been used to describe the 3 population set used for calculating the PBS score. The first letter (Y) indicates the population being analysed (YRI in this case). The CPS SNP-enriched windows are analysed for this population. The second letter (L) indicates the population to which it was compared (LWK here) and the third letter (J) indicates the outlier (JPT in this case). The number, appended with an underscore to each three letter dataset name indicates the top n^th^ percentage of PBS score cut-off used for analysis (1 = top 1%, 5 = top 5% and 10 = top 10%).

To determine whether the genomic regions enriched in CPS SNPs have an association with selective sweeps, we used two different approaches to search for possible signatures of selection in these regions. The first approach was based on the iHS (integrated Haplotype Homozygosity Score) statistic, which in principle involves the detection of unusually long haplotypes of low diversity as signatures of selection [68]. iHS scores for each SNP in the 50-SNP windows which were found to be enriched with CPS SNP were computed using the program iHS_calc [70]. For each 50 SNP window we calculated the proportion of SNPs with |iHS| > 2 which we will call iES (iHS enrichment score). The background iHS and iES score distributions were estimated on the basis of the iHS score calculated from 10,000 random contiguous 50-SNP windows or blocks for each population. Based on the background distribution, we then estimated the number of 50-SNP windows which can be expected to correspond to the top 1%, 5% and 10% of iES scores for each population. The observed number for CPS SNP-enriched windows for each population which correspond to the top 1%, 5%, and 10% iES scores were compared with the number of expected windows and the corresponding *p*-value for each observation was then estimated using bootstrap resampling. The results show that although some of the CPS SNP-enriched windows show significant iHS score enrichment, the overall distribution does not indicate any significant association of selection with these windows (Figure 5A).

One of the concerns about using a centi-morgan (cM) based physical map, such as the one used in this study, is that the signals for signatures of selection might get underestimated as the threshold of iHS > 2 used by Voight and colleagues [68] might be too stringent for a cM map based analysis. Therefore, we ran two independent sets of analysis in which the iES scores were defined on the basis of lowered thresholds of iHS > 1.75 and iHS > 1.5, respectively. However, no distinct enrichment of iHS scores was observed even in the lower threshold sets. Results from the analysis of the 5-kb windows were also found to be very similar to that obtained with the 50-SNP windows. It should, however, be kept in mind that iHS in itself might not be a very good metric for testing selective sweeps in a dataset which is known to contain many CPS SNPs of moderate allele frequencies because, unless on a single haplotype, these SNPs will have a tendency to disrupt long haplotype blocks. The results for the iHS scan, nevertheless, confirm that the CPS SNPs in CPS SNP-enriched windows show a complex distribution of SNPs which result in complex haplotype architectures, and not a single long haplotype.

To test for selective sweeps on the basis of allele frequency differentiation rather than haplotype lengths we used the population branch statistic (PBS); which has been found to be very useful in detecting high altitude adaptation-related SNPs in Tibetans relative to Han Chinese and Danish populations, as an alternative approach for detecting signatures of selective sweeps in CPS SNP-enriched windows [71]. PBS can be thought of as an estimate of the allele frequency change at a given locus in the history of a population since its divergence from another population. The idea behind this analysis is that if we consider two related populations and an outlier population, the allele frequency changes at any locus in these two populations should be equidistant (or have similar branch length) from the outlier. Therefore loci which show high allele frequency differentiation in only one of the related populations, reflected by high population branch length (and PBS score), may be potential candidates for selective sweeps.

For each population, the PBS statistic for each CPS SNP-enriched 50-SNP window was calculated using the method used by Yi et al. [69]. For the Asian populations (JPT and CHINESE) and European populations (IBS and FIN), YRI was used as the outlier population. Similarly, for the African populations YRI and LWK, the JPT population was used as the outlier. Although the choice of outlier for the populations might be questionable from a population history perspective, the distances within these populations suggest that this set can still provide reasonable estimates of branch lengths. For each 3-population set (e.g. YRI-LWK-JPT or JPT-CHB-YRI), we estimated the background distribution of the PBS scores, using 10,000 randomly-selected 50-SNP windows. We then identified score cut-offs based on the top 1%, 5% and 10% of the background distribution and estimated the number of 50-SNP windows which can be expected to be in the top 1%, 5% and 10% PBS score range for a population. The number of observed windows in the 1%, 5% and 10% range was compared to the expected number and the corresponding P-values were estimated using a bootstrap analysis. Figure 5B summarizes the PBS score distribution for the Asian and African populations. None of the windows which were found to be enriched with CPS SNPs in FIN and IBS were found to be in the top 1%, 5% or 10% range for the respective populations and hence were not retained for further analysis. It can be seen that, although some of the populations have some enrichment of high PBS scores in the CPS SNP-enriched windows, their lack of statistical significance as well as the overall distribution of PBS scores do not suggest that selection is common in these regions (Figure 5B). Although there are quite a few other tests for detecting selective sweeps [72, 73] which could have been employed for this dataset and might have identified a few more CPS SNP-enriched windows to be under selective sweeps, it is unlikely that they would change the landscape fundamentally and it can be safely concluded that selection is not the major factor causing CPS SNP enrichment in certain genomic regions. However, the efficiency of existing methodologies for detecting signatures of selection in datasets like the current 1000 Genomes dataset (which contain a large proportion low frequency SNPs, sequenced on a low coverage platform) is an important concern as genome wide variation in error rates might easily mask true signals and generate false positive signals of signature of selection. Development of parameters and efficient quality control measures well suited for identifying signatures of selection in such a dataset will significantly contribute to future work in this direction.

#### Role of recombination rate

**Figure 6:**
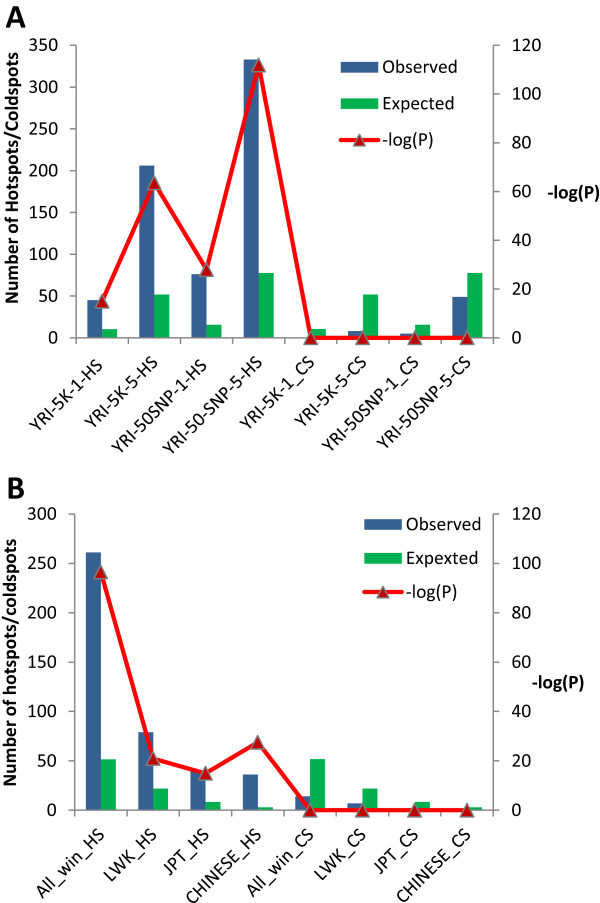
**Recombination rates in common population-specific (CPS) SNP-enriched regions. A.** The expected and observed number of hotspots (HS), defined on the basis of top 1% and 5% recombination rates) and coldspots (CS) (defined on the basis of lowest 1% and 5% recombination rates) in CPS SNP-enriched regions. **(A)** Recombination rates for the YRI was estimated on the basis of the HapMap24 YRI specific map downloaded using the UCSC table browser. The distribution of hotspots in regions detected by length based (5-kb) and window based (50-SNP) approaches using the top 1% (indicated with _1) and 5% (shown by _5) recombination rate is shown **(B)** The combined recombination map was used to identify whether the observed pattern of distribution of hotspots and cold spots in YRI also hold for JPT, LWK and CHINESE population specific windows (based on top 5% recombination rates). In addition to individual populations, the CPS SNP-enriched windows for all four populations taken together (ALL_HS and ALL_CS) are also shown.

Regions of high recombination have been shown to be related to higher SNP densities [74, 75]. As the SNP densities in the CPS SNP-enriched windows are higher in a single population compared to others, we considered whether there was any relationship between CPS SNP-enriched windows and higher recombination rates. To test the association of CPS SNP-enriched genomic regions with meiotic recombination rates, we obtained recombination hotspots based on the recombination maps generated by deCODE [74]. The distribution of recombination hotspots from the deCODE recombination map using a SRR (sex-standardized recombination rate) cut-off of 10 found only a handful of recombination hotspots within the CPS SNP-enriched regions in all populations taken together [76]. However, recombination hotspots have been found to vary significantly among populations [77, 78] and as a population-specific perspective of recombination was key for this study, in addition to the generalized deCODE recombination map, the linkage disequilibrium (LD) based HapMap YRI map (hapMapRelease24YRIRecombMap) was used to identify recombination hotspots and coldspots for the YRI population [6, 33, 34]. Similarly the combined HapMap recombination map (hapMapRelease24CombinedRecombMap) was used to identify recombination hotspots and coldspots for all other populations [6, 33, 34].We studied the genomic distribution of the recombination rates from the YRI-specific map and the genomic regions corresponding to the top 1% recombination rates were defined as recombination hotspots for YRI. A second set of hotspots, likewise, were defined on the basis of the top 5% recombination rates. Similarly, two sets of coldspots were defined by the lowest 1% and 5% recombination rates. Based on the genomic distribution of recombination rates in YRI we estimated the number of hotspot sites expected to occur in CPS SNP-enriched windows for the YRI population. The observed rates were compared with the expected rates and the statistical significance of enrichment of recombination hotspots were estimated at both 1% and 5% levels. The CPS SNP-enriched regions defined on the basis of both length (5-kb) and 50-SNP windows were analysed separately. The frequency of sites with the top 1% and 5% recombination rates in both sets of YRI-specific CPS SNP-enriched regions in comparison to the respective background distributions of genomics regions with the top 1% and 5% recombination rates has been summarized in Figure 6A. It is clear that for both kinds of windows and at both levels (top 1% and 5%) the recombination hotspots were highly enriched in the population specific SNP-enriched genomic regions. The analysis of coldspots at both 1% and 5% levels, on the other hand, show that these sites are highly under-represented in the CPS SNP-enriched regions. A similar analysis for other populations using the combined map (hapMapRelease24CombinedRecombMap) shows that the trend of very significant enrichment of these hotspots and significant depletion of the recombination coldspots is consistently seen in all populations (Figure 6B). A combined analysis of CPS SNP-enriched windows from all the populations taken together also shows the same trend (Figure 6B).

Although this analysis shows a very clear trend, as the maps used in this study are LD based, further evidence in terms of experimentally derived data for at least some of these regions will be required to reliably establish the relationship between recombination hotspots and CPS SNP-enriched windows. Nevertheless, the observed enrichment of recombination hotspots in CPS SNP-enriched genomic regions hints that high recombination might be one of the factors contributing to the generation of CPS SNP clusters. The presence of recombination hotspot(s) in a short genomic region (5 kb or 50 SNP), especially in case of a genotype based recombination map like the one used here, clearly indicates the LD architecture to be complex and the LD blocks to be short within that particular region. Moreover, as the width of a recombination hotspot (1–2 kb) is significant with respect to length of the sliding windows (5 kb or 50 SNP) used in the analysis, the presence of even a single hotspot can lower the LD of the region covered within the window considerably. The enrichment of recombination hotspots, therefore suggests that LD blocks are probably shorter and that LD is probably lower in the CPS SNP-enriched regions compared to average genomic regions. Moreover, in addition to recombination rate associated SNP density variations, the high recombination rates also suggest that the effects of population admixtures will be more prominent in these regions, which might also be an important source of the observed CPS SNP clusters. Furthermore, as recombination hotspots have been found to vary significantly among populations [77, 78]. Therefore, if recombination hotspots play a role in generating CPS SNP clusters the occurrence of these regions at different genomic positions in different populations becomes explainable.

### Functional categories and pathway distribution of CPS SNPs

**Figure 7:**
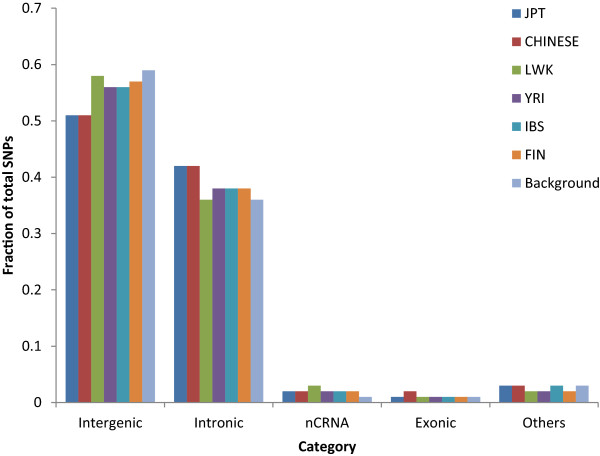
**Localization of common population-specific (CPS) SNPs in genomic regions defined on the basis of gene architecture.** The majority of the CPS SNPs were found to be intergenic and intronic. The category ncRNA includes various types of non-coding RNAs and the category “other” includes upstream, downstream and UTR SNPs. The expected distribution based on overall occurrence of SNPs in human genome is shown as “Background”.

To study the functional relevance of the CPS SNPs we analysed their localization with respect to known genes. As seen in the case of most novel variants identified by the 1000 Genomes project [17], as well as what can be expected on the basis of the background distribution of SNPs, most of the CPS SNPs were found to be either intergenic or intronic (Figure 7). Despite certain minor variations, for example in FIN and JPT, the overall distribution of the CPS SNPs in different major genomic regions was observed to be similar in all the populations. Interestingly however, the number of coding non-synonymous CPS SNPs in these populations (Table 4) were found be independent of the total number of CPS SNPs in them. These coding non-synonymous CPS SNPs were found to occur in roughly equal numbers in YRI, LWK and JPT, only a single CPS SNP was detected in the IBS, and were missing in the FIN and CHINESE populations. The functional impact of these non-synonymous coding CPS SNPs was assessed using a combination of four different SNP function prediction tools (SIFT, Polyphen 2, LRT, Mutation taster) which predicted most of these SNPs to have a potential functional impact [79–82]. The list of coding non-synonymous SNPs along with their predicted functional significance is summarized in Table 4.

**Table 4:** **Coding non-synonymous common population-specific SNPs and potential functional impact**

Pop	SNP	Gene	SIFT	PolyPhen-2	LRT	Mutation Taster
IBS	rs34804805	MRPL35	T	B	N	N
JPT	rs3749130	ARHGAP25	D	P	N	N
	rs2296151	ASIP	T	P	N	N
	rs17846992	CCKAR	D	D	N	D
	rs77945315	CSNK1E	D	B	D	D
	rs76875855	KRT73	D	D	D	N
	rs1800885	MTNR1A	T	P	U	D
	rs41428447	NDUFS2	D	B	D	D
	rs74548274	OR5D13	T	P	U	N
LWK	rs61749435	ABCA4	D	B	D	N
	rs34018205	ATP8B1	T	B	D	D
	rs34744783	C20orf26	D	B	N	N
	rs34347250	EGLN3	T	B	D	D
	rs6413484	GHR	D	B	N	N
	rs34752664	KCNF1	T	B	D	N
	rs35706839	MCCC1	T	NA	D	D
	rs76085152	NLRP12	D	NA	N	N
	rs104895564	NLRP12	T	D	N	N
	rs35651739	NOXO1	D	D	N	N
	rs3087400	REV1	T	B	N	N
	rs34994431	SLC16A11	T	D	N	D
YRI	rs35755269	DIAPH1	NA	NA	N	P
	rs34901743	HDAC3	T	D	D	P
	rs1065759	HLCS	T	P	N	D
	rs6299	HTR1D	D	P	N	P
	rs8176804	PAWR	T	B	N	N
	rs34781001	RPN1	T	P	D	D
	rs2229464	TGM1	T	B	N	D
	rs59896509	TRIM5	D	D	D	D
	rs1799126	UPK3B	D	NA	NA	N
	rs1799125	UPK3B	T	NA	U	N
	rs34995077	ZNF565	T	P	N	N

Functions were assessed using a set of four different tools [79–82]. The predictions D and T for SIFT mean Deleterious and Tolerable respectively. For Polyphen2, B = Benign; P = Possibly Damaging; D = Probably Damaging and NA refers to SNPs for which no information was found. Similarly for LRT; D = Deleterious Non-synonymous SNP; N = Neutral; U = Unknown and for MutationTaster; N = Polymorphism; D = Disease Causing; P = Polymorphism automatic.

Eleven coding non-synonymous CPS SNPs were observed in the YRI mapping to10 different genes, 8 of them were predicted to be functional by at least one of the tools. Four of the 10 CPS SNPs containing genes were detected to have known association with a disease, includin [79, 80]g *HLCS* (holocarboxylase synthetase deficiency), *TGM1* (congenital ichthyosis), *DIAPh1* (deafness), and *PAWR* (which induces apoptosis in certain cancer cells). Moreover, a functional SNP was detected in *TRIM5* which is a capsid-specific restriction factor involved in blocking viral replication early in the life cycle. Additionally, two coding non-synonymous SNPs were detected in the *UPK3B* gene which plays an important role in AUM-cytoskeleton interaction in terminally differentiated urothelial cells.

In the LWK population 12 coding non-synonymous CPS SNPs in 11 genes were observed, 5 of which are linked to disease phenotypes. These include *ABCA4*, linked to Stargardt disease 1, hereditary macular degeneration and retinitis pigmentosa; *ATP8B1*, associated with various forms of cholestasis, GHR, which is linked to Laron syndrome, resulting in growth impairment; *MCCC1*, involved in methylcrotonoyl-CoA carboxylase 1 deficiency, and two SNPs in *NLRP12* gene, which is associated with familial cold autoinflammatory syndrome. In the JPT population 8 non-synonymous CPS SNPs, all of which were predicted to be functional, were observed in 8 genes. Some of these genes were found to be involved in melatonin activity, melanogenesis, olfaction and hair formation. Only a single non-synonymous CPS SNP was detected in the *MRP35* gene in the IBS population, whereas none was found to occur in the CHINESE and the FIN populations.

Additionally, a total of 520 CPS SNPs with probable consequences for gene regulation, all from RegulomeDB category 2, which demonstrates direct evidence of a binding through ChIP-seq and DNase data with either a matched position weight matrix to the ChIP-seq factor or a DNase footprint, were identified (Additional file 4) [83]. Of the putative regulatory variants identified, the majority are intergenic (234) and intronic (224). Approximately 3 times as many upstream (24) compared to downstream (7) variants were identified, while 3′-UTR variants were approximately double the number in the 5′-UTR. The occurrence of these potential regulatory SNPs, in addition to the potentially functional coding non-synonymous CPS SNPs indicate that in spite of occurring in a single population, at least some of the CPS SNPs might play a significant functional role in some of these populations.

**Figure 8:**
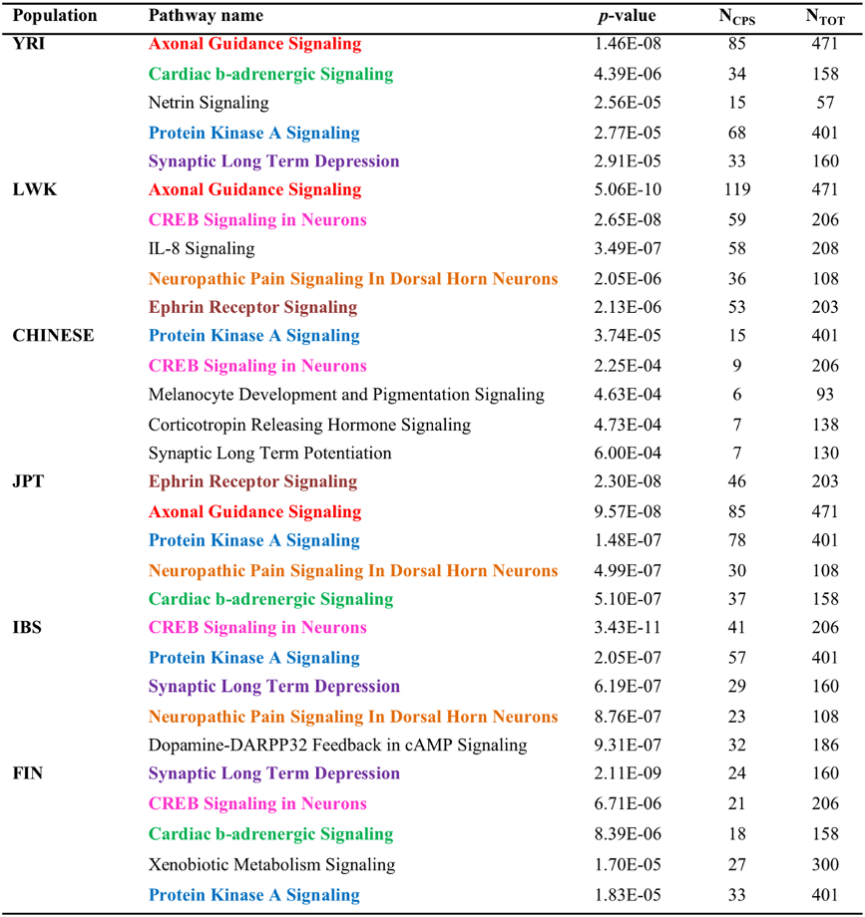
**Ingenuity canonical pathways enriched with common population-specific (CPS) SNPs.** The 5 most overrepresented pathways for each population identified using IPA are shown. N_CPS_ denotes the number of CPS SNP containing genes in the pathway and N_TOT_ denotes the total number of genes in the pathway. Each pathway which was found to occur in two or more populations is shown in bold and a distinct colour.

To identify possible functional preference in the distribution of CPS SNPs in different populations we used the Ingenuity Pathway Analysis tool (IPA) [84] and DAVID [85] to identify functional classes, metabolic pathways and regulatory networks enriched in CPS SNPs in each population. The populations CEU, GBR and TSI, were excluded from this analysis as they contain too few CPS SNPs for generating statistically and biologically meaningful results. The top 5 canonical pathways found to be overrepresented in the CPS SNPs for each population using IPA are shown in Figure 8. We also prepared an extended gene list for each population which, in addition to genes for coding and intronic SNPs included nearby genes for the intergenic SNPs. This set was created to provide a more inclusive view of the functional preference as intergenic SNPs which form large proportion of CPS SNPs, are completely excluded from the pathway analysis. The top 5 CPS SNP-enriched canonical pathways for each population derived using the extended gene set are tabulated in Additional file 5. As expected, the pathways that were found using both the approaches show a significant overlap. Interestingly, there was a very significant overlap in pathways that were detected to be enriched in CPS SNPs between different populations. We also performed an analysis for enrichment of regulatory networks in the CPS SNPs and their corresponding genes. Regulatory networks overrepresented in (a) CPS SNP containing genes and (b) extended gene list (list of all genes containing variants, as well as nearest neighbour genes for intergenic variants), for each population are summarized in Additional file 6 which also exhibited significant overlap between different populations.

Using DAVID, we identified a number of CPS SNP-enriched disease, pathway, and gene ontology (GO) classes for each population. As observed for the pathways detected using IPA, the CPS SNP-enriched disease, pathway and GO classes identified using DAVID overlapped between the different populations (Additional file 7). Moreover, the pathways identified using DAVID in many cases supported the pathways identified using the IPA tool. One of the major functional classes/pathways, which were observed to show significant CPS SNP enrichment in most of the populations and in multiple analyses, was the axon guidance signalling or axonogenesis pathway. This observation also supports previous work where genetic variations in genes involved in axon guidance signalling have been found to show significantly high levels of population differentiation [86, 87]. Moreover, a recent study aimed at identifying loci under parallel divergence (loci that have undergone moderate allele frequency changes in multiple independent human lineages) found most parallel divergent genes to occur in this pathway [88]. This may explain our observation for CPS SNP enrichment in the corresponding genomic regions in multiple populations. It is also interesting to note that several recent studies have shown this pathway to be one of the major mutational targets in pancreatic and other cancers [89–91]. It would be an interesting follow up study to probe whether evolutionary forces, like mutation rate, might contribute to the observed SNP accumulation in regions where genes for these pathways occur and whether this enrichment has any adaptive relevance. Similar overlap was observed in many other CPS SNP-enriched pathways including protein kinase A signalling and CREB Signaling in Neurons (Figure 8), which points to underlying functional similarities in the distribution of CPS SNPs in different populations.

Current functional and pathway analysis is clearly limited by the state of current knowledge about gene interactions and functions. Well studied genes and pathways tend to contain more complete, validated interaction and functional data in contrast to less studied genes and pathways are. As the information around functional gene networks and regulatory pathways increases, we can anticipate that there may be additional gene functions and networks that are identified as being differentially regulated between populations; so these results can only represent our findings with respect to the current state of knowledge

## Conclusions

In this study we have highlighted some interesting observations with regard to population-specific genetic variation, using an unbiased data set generated by whole genome sequencing. Firstly, we showed that CPS SNPs are abundant but are not randomly distributed and can cluster into regions that can span up to several kilobases. Secondly we have illustrated that at least some of the CPS SNPs are likely to have a phenotypic or functional impact. Thirdly, in terms of mechanism, we were unable to detect any evidence for selection in the regions of high CPS SNP density but interestingly, these regions more often associate with regions of high recombination. The enrichment of recombination hotspots in a way also indicates that the LD in the CPS SNP-enriched region is lower than that in the average genome and rules out any possible role of LD in generating CPS enriched regions. Finally, functional enrichment analysis of the CPS SNPs and their associated genes has highlighted some interesting pathways and functions over represented in several populations. Particularly, it highlighted possible hyper mutability of genes involved in axonal guidance signalling perhaps suggesting some evolutionary plasticity in this pathway.

Avenues for future exploration have been highlighted. However, there are several caveats. Firstly, the number of individuals per population for whom we have full genome sequences is presently low (N < 100). Secondly, the definition of a population in terms of origin and admixture is at times vague and increased mobility worldwide leads to elevated levels of admixture. Moreover, the numbers of variants analysed is only a small subset (<1%) of all population-specific variants since rare variants (MAF < 0.05) have not been included. Genome sequencing of global populations is providing data which will assist in teasing out ancestral populations and will shed further light on population differentiation and adaptation. The availability of more extensive data along with an increased depth of sequencing, which permits the reliable study of rare genetic variants and structural variants, is therefore required for a better understanding of the relationship between unique genotypic variations and their geographical contexts.

## Methods

### Data retrieval and processing

The recent version (Phase1, version 3, October 2012) of the 1000 Genomes vcf files containing phased genotypes for 36.7 million autosomal SNPs and 1.38 Million autosomal SSVs were downloaded from 1000 Genomes Project ftp server [92]. The ancestral allele information for SNPs on the basis of multi species alignments, for all variants was also downloaded from the 1000 Genomes ftp site. The conversion of the 1000 Genomes data to PLINK format was performed using the VCF tools [93, 94]. Frequency calculations and many other data manipulation operations were performed using PLINK [94]. The admixed populations (ASW, CLM, MXL and PUR) were excluded and the Chinese populations (CHB and CHS) were merged into a single population using PLINK which we refer to as “CHINESE”. The SNPs were classified as common in a population if the MAF was observed to be greater than 0.05 in that population. SNPs with lower MAF were treated as rare.

### Genomic distribution and regional enrichment analysis

Identification of enrichment of CPS SNPs in genomic regions was performed using custom Perl scripts. We used two sliding window based approaches. In the first approach, each chromosome was scanned using sliding and non-overlapping 50-SNP windows and the frequency of CPS SNPs in each window was computed. Based on the overall occurrence of CPS SNPs in the entire chromosome the cumulative hypergeometric *p*-value for enrichment of CPS SNPs in each window was estimated. To correct for multiple hypothesis testing we used a conservative *p*-value cut-off of <5 × 10 ^-8^ for the identification of windows enriched with CPS SNPs. In the second approach we employed a similar scan using 5-kb non-overlapping windows.

### Selection scan

Signatures of selection were evaluated using two different approaches. The haplotype homozygosity based iHS score was calculated using the WHAMM package [95]. As calculation of iHS requires physical positions to be specified, we downloaded the combined linkage physical map for human genome build GrCh37 from Rutgers Map [96] and incorporated the physical positions into the existing data. For each population, iHS scores for SNPs occurring in the 50-SNP windows which were found to be enriched in CPS SNPs in that population were calculated using the iHS_calc script from the WHAMM package. To estimate the background iHS distribution for each population, we randomly sampled 10,000 50-SNP blocks and calculated the iHS scores for the SNPs occurring in these blocks. Based on allele frequency bins derived from the background, the iHS scores were then standardized. As an extension of the iHS scores we also defined iHS enrichment scores (iES) scores which is the proportion of SNPs in each 50-SNP window which has |iHS| > 2. Windows showing the top 1%, 5% and 10% iES scores were respectively selected as three levels for the analysis. For each level the expected iES distribution in all CPS SNP windows of a population was estimated and compared to the actual distribution. Statistical significance of overrepresentation of iES scores in CPS SNP-enriched windows of a population was estimated using a *p*-value calculated by a bootstrap resampling analysis. A similar analysis was also performed for CPS SNP-enriched 5-kb windows in each population. In addition, a separate set of analyses were performed for both 50-SNP and 5-kb windows, considering only SNPs with a minimum MAF of 0.05.

The calculation of PBS was carried out following the methods proposed by Yi and colleagues [71]. For calculating PBS scores for the African populations (YRI and LWK), JPT was used as an outlier. For the Asian populations CHINESE and JPT, YRI was used as an outlier. Similarly for the European populations (FIN and IBS) YRI was used as the outlier. For each three population set (like YRI-LWK-JPT or JPT-CHB-YRI) we estimated the background distribution of the PBS scores, using 10,000, randomly selected 50-SNP windows. We then identified score cut-offs based on the top 1%, 5% and 10% of the background distribution and estimated the number of 50-SNP and 5-kb windows which can be expected to be in the top 1% ,5% and 10% PBS score range for a population. The number of observed windows in the 1%, 5% and 10% range was compared to the expected number and the corresponding *p*-values were estimated using a bootstrap analysis.

### Recombination rate

We retrieved the deCODE recombination map and the HapMap related recombination maps (hapMapRelease24YRIRecombMap and hapMapRelease24CombinedRecombMap) using the UCSC table browser [97]. The distribution of recombination hotspots from the deCODE recombination map using a SRR (sex-standardized recombination rate) cut-off of 10 found only a few hotspots in the gene set and were not analysed further.

The HapMap YRI recombination map (hapMapRelease24YRIRecombMap) was used to identify recombination hotspots and coldspots in YRI and the combined dataset. The distribution of recombination rates was studied to select genomic regions showing the top 1% recombination rate scores and these regions were designated as recombination hotspots. We also used the top 5% recombination rate scores to select a second set of hotspots. Similarly, the two sets of coldspots likewise were defined by the lowest 1% and 5% recombination rates. Based on the genomic distribution of recombination rates in YRI (hapMapRelease24YRIRecombMap) we estimated the number of hotspot sites expected to occur in CPS SNP-enriched windows for YRI. The expected value was compared to the observed value and a cumulative hypergeometric *p*-value was used to estimate the statistical significance of the over and underrepresentation for recombination hotspots and coldspots in the CPS SNP-enriched 50-SNP windows and the CPS SNP enriched 5 kb-windows in YRI. Similar analyses were conducted for all other populations, individually as well as combined together, using the HapMap combined recombination map (hapMapRelease24CombinedRecombMap).

### SNP function assessment

The genomic contexts of all CPS SNPs were determined using ANNOVAR [98], which was also used to annotate potentially functional non-synonymous variants based on their predicted functional impact at the protein level. ANNOVAR derives pre-computed functional impact scores for SIFT [80], POLYPHEN2 [79] *,* LRT [82] and Mutation Taster [81]. Non-synonymous variants were considered to have a functional impact if the recommended score criteria for any one of the algorithms were met, SIFT: ≥ 0.95, POLYPHEN2: ≥ 0.85, LRT ≥ 0.5, Mutation Taster ≥ 0.50.

In order to identify non-coding CPS SNPs that may have an effect on the binding of regulatory factors, intronic variants and those flanking genes were searched against the RegulomeDB database [83], which employs a heuristic scoring system based on the confidence that the variant lies in a regulatory element and whether it has known or possible functional consequences such as alteration of Transcript Factor (TF) binding and changes in expression patterns of the associated gene(s). dbSNP [99] variants are classified into 6 categories, with category 1 having highest confidence due to associated eQTL data, and category 6 the lowest. Only CPS SNPs belonging to categories 1 and 2 were considered to be regulation-modifying, since they are the most likely to result in a functional consequence.

### IPA analysis

For each population, two gene lists were generated from the CPS SNP set. The first contained only genes that included selected variants, identified by rs IDs [99]. The second contained all genes that contained the identified SNPs, as well as nearest neighbour genes for the SNPs that were intergenic. By definition, the second list contained more genes than the first. Ingenuity Pathway Analysis (IPA) software was used to analyse gene interaction networks in the gene lists, as well as enriched ‘canonical’ pathways describing well characterised and validated regulatory pathways [84].

### DAVID analysis

The Database for Annotation, Visualisation and Integrated Discovery (DAVID) [85] is an online tool that accepts a list of genes as input and performs functional analysis on them. It provides a list of functions enriched in the gene list, and clusters these functions according to their similarity. Functions include gene ontology (GO) and Swiss-Prot annotation, InterPro matches, OMIM [100] and other disease links, as well as KEGG [101, 102] and other pathway database links. The gene-enrichment analysis is based on the Fisher’s Exact test, which determines whether or not a given list of genes is enriched for a certain function label or if this function occurs in the list by chance. A *p*-value shows the significance and adjusted *p*-values are also provided, after correction for multiple testing. The gene lists for each population that contained CPS SNPs were run through DAVID to identify overrepresented pathways and other functional labels.

### Function and disease association of CPS-SNP containing genes

Potential functions of CPS-SNP containing genes and their role in various diseases were inferred from the GeneCards database [103].

## Electronic supplementary material

Additional file 1: **List of CPS SNPs for each population according to the 9 population model.** (XLSX 7 MB)

Additional file 2: **50-SNP windows detected to be enriched with CPS SNPs.** (XLSX 80 KB)

Additional file 3: **5-kb windows detected to be enriched with CPS SNPs.** (XLSX 72 KB)

Additional file 4: **Potentially regulatory CPS SNPs.** (XLSX 33 KB)

Additional file 5: **IPA pathways enriched in CPS SNP associated genes.** (XLSX 12 KB)

Additional file 6: **Regulatory networks enriched in CPS SNP associated genes.** (XLSX 11 KB)

Additional file 7: **Functional classes enriched with CPS SNP associated genes identified using DAVID.** (XLSX 393 KB)

## Abbreviations

SNP: Single nucleotide polymorphism
SSV: Short structural variant
MAF: Minor allele frequency
CPS: Common population-specific
RPS: Rare population-specific
iHS: Integrated haplotype homozygosity score
iES: iHS enrichment score
PBS: Population branch statistic
SRR: Sex-standardized recombination rate
YRI: Yoruba in Ibadan, Nigeria
LWK: Luhya in Webuye, Kenya
JPT: Japanese in Tokyo, Japan
CHB: Han Chinese in Beijing, China
CHS: Han Chinese South
MXL: Mexican ancestry in Los Angeles, CA, USA
PUR: Puerto Ricans in Puerto Rico
CLM: Colombians in Medellín, Colombia
IBS: Berian populations in Spain
GBR: British from England and Scotland
CEU: Utah residents with ancestry from northern and western Europe
FIN: Finnish in Finland
TSI: Toscani in Italia
ASW: African ancestry in SW USA
LD: Linkage disequilibrium
GO: Gene ontology.

## References

[1] Barbujani G, Colonna V (2010). Human genome diversity: frequently asked questions. Trends Genet.

[2] Henn BM, Cavalli-Sforza LL, Feldman MW (2012). The great human expansion. Proc Natl Acad Sci.

[3] Balaresque PL, Ballereau SJ, Jobling MA (2007). Challenges in human genetic diversity: demographic history and adaptation. Hum Mol Genet.

[4] Scheinfeldt LB, Tishkoff SA (2013). Recent human adaptation: genomic approaches, interpretation and insights. Nat Rev Genet.

[5] Hancock AM, Witonsky DB, Alkorta-Aranburu G, Beall CM, Gebremedhin A, Sukernik R, Utermann G, Pritchard JK, Coop G, Di Rienzo A (2010). Adaptations to climate-mediated selective pressures in humans. PLoS Genet.

[6] The International HapMap Consortium (2005). A haplotype map of the human genome. Nature.

[7] Rosenberg NA, Pritchard JK, Weber JL, Cann HM, Kidd KK, Zhivotovsky LA, Feldman MW (2002). Genetic structure of human populations. Science (80- ).

[8] Li JZ, Absher DM, Tang H, Southwick AM, Casto AM, Ramachandran S, Cann HM, Barsh GS, Feldman M, Cavalli-Sforza LL, Myers RM (2008). Worldwide human relationships inferred from genome-wide patterns of variation. Science (80- ).

[9] Hinds DA, Stuve LL, Nilsen GB, Halperin E, Eskin E, Ballinger DG, Frazer KA, Cox DR (2005). Whole-genome patterns of common DNA variation in three human populations. Science (80- ).

[10] Nelson MR, Bryc K, King KS, Indap A, Boyko AR, Novembre J, Briley LP, Maruyama Y, Waterworth DM, Waeber G, Vollenweider P, Oksenberg JR, Hauser SL, Stirnadel HA, Kooner JS, Chambers JC, Jones B, Mooser V, Bustamante CD, Roses AD, Burns DK, Ehm MG, Lai EH (2008). The population reference sample, POPRES: a resource for population, disease, and pharmacological genetics research. Am J Hum Genet.

[11] Jakobsson M, Scholz SW, Scheet P, Gibbs JR, VanLiere JM, Fung H-C, Szpiech ZA, Degnan JH, Wang K, Guerreiro R, Bras JM, Schymick JC, Hernandez DG, Traynor BJ, Simon-Sanchez J, Matarin M, Britton A, van de Leemput J, Rafferty I, Bucan M, Cann HM, Hardy JA, Rosenberg NA, Singleton AB (2008). Genotype, haplotype and copy-number variation in worldwide human populations. Nature.

[12] Novembre J, Ramachandran S (2011). Perspectives on human population structure at the cusp of the sequencing era. Annu Rev Genomics Hum Genet.

[13] Theunert C, Tang K, Lachmann M, Hu S, Stoneking M (2012). Inferring the history of population size change from genome-wide SNP data. Mol Biol Evol.

[14] Moorjani P, Patterson N, Hirschhorn JN, Keinan A, Hao L, Atzmon G, Burns E, Ostrer H, Price AL, Reich D (2011). The history of African gene flow into Southern Europeans, Levantines, and Jews. PLoS Genet.

[15] Albrechtsen A, Nielsen FC, Nielsen R (2010). Ascertainment biases in SNP chips affect measures of population divergence. Mol Biol Evol.

[16] Li Y, Vinckenbosch N, Tian G, Huerta-Sanchez E, Jiang T, Jiang H, Albrechtsen A, Andersen G, Cao H, Korneliussen T, Grarup N, Guo Y, Hellman I, Jin X, Li Q, Liu J, Liu X, Sparso T, Tang M, Wu H, Wu R, Yu C, Zheng H, Astrup A, Bolund L, Holmkvist J, Jorgensen T, Kristiansen K, Schmitz O, Schwartz TW (2010). Resequencing of 200 human exomes identifies an excess of low-frequency non-synonymous coding variants. Nat Genet.

[17] Abecasis GR, Altshuler D, Auton A, Brooks LD, Durbin RM, Gibbs RA, Hurles ME, McVean GA (2010). A map of human genome variation from population-scale sequencing. Nature.

[18] The 1000 Genomes Project Consortium (2012). An integrated map of genetic variation from 1,092 human genomes. Nature.

[19] Tennessen JA, Bigham AW, O’Connor TD, Fu W, Kenny EE, Gravel S, McGee S, Do R, Liu X, Jun G, Kang HM, Jordan D, Leal SM, Gabriel S, Rieder MJ, Abecasis G, Altshuler D, Nickerson DA, Boerwinkle E, Sunyaev S, Bustamante CD, Bamshad MJ, Akey JM, Broad GO, Seattle GO, on behalf of the NESP (2012). Evolution and functional impact of rare coding variation from deep sequencing of human exomes. Science (80- ).

[20] Green RE, Krause J, Briggs AW, Maricic T, Stenzel U, Kircher M, Patterson N, Li H, Zhai W, Fritz MH-Y, Hansen NF, Durand EY, Malaspinas A-S, Jensen JD, Marques-Bonet T, Alkan C, PrÃ¼fer K, Meyer M, Burbano HA, Good JM, Schultz R, Aximu-Petri A, Butthof A, HÃ¶ber B, HÃ¶ffner B, Siegemund M, Weihmann A, Nusbaum C, Lander ES, Russ C (2010). A draft sequence of the neandertal genome. Science (80- ).

[21] Reich D, Green RE, Kircher M, Krause J, Patterson N, Durand EY, Viola B, Briggs AW, Stenzel U, Johnson PLF, Maricic T, Good JM, Marques-Bonet T, Alkan C, Fu Q, Mallick S, Li H, Meyer M, Eichler EE, Stoneking M, Richards M, Talamo S, Shunkov MV, Derevianko AP, Hublin J-J, Kelso J, Slatkin M, Paabo S (2010). Genetic history of an archaic hominin group from Denisova Cave in Siberia. Nature.

[22] Alves I, Sramkova Hanulova A, Foll M, Excoffier L (2012). Genomic data reveal a complex making of humans. PLoS Genet.

[23] Hammer MF, Woerner AE, Mendez FL, Watkins JC, Wall JD (2011). Genetic evidence for archaic admixture in Africa. Proc Natl Acad Sci.

[24] Lachance J, Vernot B, Elbers CC, Ferwerda B, Froment A, Bodo J-M, Lema G, Fu W, Nyambo TB, Rebbeck TR, Zhang K, Akey JM, Tishkoff SA (2012). Evolutionary history and adaptation from high-coverage whole-genome sequences of diverse African hunter-gatherers. Cell.

[25] Slatkin M (2000). Allele age and a test for selection on rare alleles. Philos Trans R Soc London Ser B Biol Sci.

[26] Gibson G (2012). Rare and common variants: twenty arguments. Nat Rev Genet.

[27] Kryukov GV, Pennacchio LA, Sunyaev SR (2007). Most rare missense alleles are deleterious in humans: implications for complex disease and association studies. Am J Hum Genet.

[28] Yang J, Benyamin B, McEvoy BP, Gordon S, Henders AK, Nyholt DR, Madden PA, Heath AC, Martin NG, Montgomery GW, Goddard ME, Visscher PM (2010). Common SNPs explain a large proportion of the heritability for human height. Nat Genet.

[29] Hartford CM, Duan S, Delaney SM, Mi S, Kistner EO, Lamba JK, Huang RS, Dolan ME (2009). Population-specific genetic variants important in susceptibility to cytarabine arabinoside cytotoxicity. Blood.

[30] Prescott NJ, Dominy KM, Kubo M, Lewis CM, Fisher SA, Redon R, Huang N, Stranger BE, Blaszczyk K, Hudspith B, Parkes G, Hosono N, Yamazaki K, Onnie CM, Forbes A, Dermitzakis ET, Nakamura Y, Mansfield JC, Sanderson J, Hurles ME, Roberts RG, Mathew CG (2010). Independent and population-specific association of risk variants at the IRGM locus with Crohn’s disease. Hum Mol Genet.

[31] Parra EJ, Marcini A, Akey J, Martinson J, Batzer MA, Cooper R, Forrester T, Allison DB, Deka R, Ferrell RE, Shriver MD (1998). Estimating African American admixture proportions by use of population-specific alleles. Am J Hum Genet.

[32] Lohmueller KE, Bustamante CD, Clark AG (2010). The effect of recent admixture on inference of ancient human population history. Genetics.

[33] The International HapMap Consortium (2003). The international HapMap project. Nature.

[34] The International HapMap Consortium (2007). A second generation human haplotype map of over 3.1 million SNPs. Nature.

[35] Baye TM, Wilke RA, Olivier M (2009). Genomic and geographic distribution of private SNPs and pathways in human populations. Per Med.

[36] Fu W, O’Connor TD, Jun G, Kang HM, Abecasis G, Leal SM, Gabriel S, Altshuler D, Shendure J, Nickerson DA, Bamshad MJ, Project NES, Akey JM (2013). Analysis of 6,515 exomes reveals the recent origin of most human protein-coding variants. Nature.

[37] Chen J, Zheng H, Bei J-X, Sun L, Jia W, Li T, Zhang F, Seielstad M, Zeng Y-X, Zhang X, Liu J (2009). Genetic structure of the Han Chinese population revealed by genome-wide SNP variation. Am J Hum Genet.

[38] Qin P, Li Z, Jin W, Lu D, Lou H, Shen J, Jin L, Shi Y, Xu S (2013). A panel of ancestry informative markers to estimate and correct potential effects of population stratification in Han Chinese. Eur J Hum Genet.

[39] Xu S, Yin X, Li S, Jin W, Lou H, Yang L, Gong X, Wang H, Shen Y, Pan X, He Y, Yang Y, Wang Y, Fu W, An Y, Wang J, Tan J, Qian J, Chen X, Zhang X, Sun Y, Zhang X, Wu B, Jin L (2009). Genomic dissection of population substructure of Han Chinese and its implication in association studies. Am J Hum Genet.

[40] Murray T, Beaty TH, Mathias RA, Rafaels N, Grant AV, Faruque MU, Watson HR, Ruczinski I, Dunston GM, Barnes KC (2010). African and non-African admixture components in African Americans and an African Caribbean population. Genet Epidemiol.

[41] Campbell MC, Tishkoff SA (2010). The evolution of human genetic and phenotypic variation in Africa. Curr Biol.

[42] Tishkoff SA, Reed FA, Friedlaender FR, Ehret C, Ranciaro A, Froment A, Hirbo JB, Awomoyi AA, Bodo J-M, Doumbo O, Ibrahim M, Juma AT, Kotze MJ, Lema G, Moore JH, Mortensen H, Nyambo TB, Omar SA, Powell K, Pretorius GS, Smith MW, Thera MA, Wambebe C, Weber JL, Williams SM (2009). The genetic structure and history of Africans and African Americans. Science (80-).

[43] De Filippo C, Bostoen K, Stoneking M, Pakendorf B (2012). Bringing together linguistic and genetic evidence to test the Bantu expansion. Proc R Soc B Biol Sci.

[44] Joubert BR, North KE, Wang Y, Mwapasa V, Franceschini N, Meshnick SR, Lange EM (2010). Comparison of genome-wide variation between Malawians and African ancestry HapMap populations. J Hum Genet.

[45] Nakaoka H, Mitsunaga S, Hosomichi K, Shyh-Yuh L, Sawamoto T, Fujiwara T, Tsutsui N, Suematsu K, Shinagawa A, Inoko H, Inoue I (2013). Detection of ancestry informative HLA alleles confirms the admixed origins of Japanese population. PLoS One.

[46] Hanihara K (1991). Dual structure model for the population history of the Japanese. Japan Rev.

[47] Yamaguchi-Kabata Y, Tsunoda T, Kumasaka N, Takahashi A, Hosono N, Kubo M, Nakamura Y, Kamatani N (2012). Genetic differences in the two main groups of the Japanese population based on autosomal SNPs and haplotypes. J Hum Genet.

[48] Ralph P, Coop G (2013). The geography of recent genetic ancestry across Europe. PLoS Biol.

[49] Novembre J, Johnson T, Bryc K, Kutalik Z, Boyko AR, Auton A, Indap A, King KS, Bergmann S, Nelson MR, Stephens M, Bustamante CD (2008). Genes mirror geography within Europe. Nature.

[50] Palo JU, Ulmanen I, Lukka M, Ellonen P, Sajantila A (2009). Genetic markers and population history: Finland revisited. Eur J Hum Genet.

[51] Salmela E, Lappalainen T, Fransson I, Andersen PM, Dahlman-Wright K, Fiebig A, Sistonen P, Savontaus M-L, Schreiber S, Kere J, Lahermo P¤ (2008). Genome-wide analysis of single nucleotide polymorphisms uncovers population structure in Northern Europe. PLoS One.

[52] Chuang JH, Li H (2004). Functional bias and spatial organization of genes in mutational hot and cold regions in the human genome. PLoS Biol.

[53] Lindblad-Toh K, Winchester E, Daly MJ, Wang DG, Hirschhorn JN, Laviolette J-P, Ardlie K, Reich DE, Robinson E, Sklar P, Shah N, Thomas D, Fan J-B, Gingeras T, Warrington J, Patil N, Hudson TJ, Lander ES (2000). Large-scale discovery and genotyping of single-nucleotide polymorphisms in the mouse. Nat Genet.

[54] Tenaillon MI, Austerlitz F, Tenaillon O (2008). Apparent mutational hotspots and long distance linkage disequilibrium resulting from a bottleneck. J Evol Biol.

[55] Sainudiin R, Clark A, Durrett R (2007). Simple models of genomic variation in human SNP density. BMC Genomics.

[56] McDonald MJ, Wang W-C, Huang H-D, Leu J-Y (2011). Clusters of nucleotide substitutions and insertion/deletion mutations are associated with repeat sequences. PLoS Biol.

[57] Amos W (2010). Even small SNP clusters are non-randomly distributed: is this evidence of mutational non-independence?. Proc R Soc B Biol Sci.

[58] Hofer T, Foll M, Excoffier L (2012). Evolutionary forces shaping genomic islands of population differentiation in humans. BMC Genomics.

[59] Carlson CS, Thomas DJ, Eberle MA, Swanson JE, Livingston RJ, Rieder MJ, Nickerson DA (2005). Genomic regions exhibiting positive selection identified from dense genotype data. Genome Res.

[60] Weir BS, Cardon LR, Anderson AD, Nielsen DM, Hill WG (2005). Measures of human population structure show heterogeneity among genomic regions. Genome Res.

[61] Schmid K, Yang Z (2008). The trouble with sliding windows and the selective pressure in BRCA1. PLoS One.

[62] Pasanen MK, Neuvonen PJ, Niemi M (2007). Global analysis of genetic variation in SLCO1B1. Pharmacogenomics.

[63] Mwinyi J, Köpke K, Schaefer M, Roots I, Gerloff T (2008). Comparison of SLCO1B1 sequence variability among German, Turkish, and African populations. Eur J Clin Pharmacol.

[64] Turner TL, Hahn MW (2007). Locus- and population-specific selection and differentiation between incipient species of anopheles gambiae. Mol Biol Evol.

[65] Harr B (2006). Genomic islands of differentiation between house mouse subspecies. Genome Res.

[66] Myles S, Tang K, Somel M, Green RE, Kelso J, Stoneking M (2008). Identification and analysis of genomic regions with large between-population differentiation in humans. Ann Hum Genet.

[67] Hofer T, Ray N, Wegmann D, Excoffier L (2009). Large allele frequency differences between human continental groups are more likely to have occurred by drift during range expansions than by selection. Ann Hum Genet.

[68] Nielsen R, Hellmann I, Hubisz M, Bustamante C, Clark AG (2007). Recent and ongoing selection in the human genome. Nat Rev Genet.

[69] Feder JL, Nosil P (2010). **The efficacy of divergence hitchhiking in generating genomic islands during ecological speciation**. Evolution (N Y).

[70] Voight BF, Kudaravalli S, Wen X, Pritchard JK (2006). A map of recent positive selection in the human genome. PLoS Biol.

[71] Yi X, Liang Y, Huerta-Sanchez E, Jin X, Cuo ZXP, Pool JE, Xu X, Jiang H, Vinckenbosch N, Korneliussen TS, Zheng H, Liu T, He W, Li K, Luo R, Nie X, Wu H, Zhao M, Cao H, Zou J, Shan Y, Li S, Yang Q, Asan, Ni P, Tian G, Xu J, Liu X, Jiang T, Wu R (2010). Sequencing of 50 human exomes reveals adaptation to high altitude. Science (80- ).

[72] Sabeti PC, Varilly P, Fry B, Lohmueller J, Hostetter E, Cotsapas C, Xie X, Byrne EH, McCarroll SA, Gaudet R, Schaffner SF, Lander ES (2007). Genome-wide detection and characterization of positive selection in human populations. Nature.

[73] Chen H, Patterson N, Reich D (2010). Population differentiation as a test for selective sweeps. Genome Res.

[74] Duret L, Arndt PF (2008). The impact of recombination on nucleotide substitutions in the human genome. PLoS Genet.

[75] Hellmann I, Prüfer K, Ji H, Zody MC, Pääbo S, Ptak SE (2005). Why do human diversity levels vary at a megabase scale?. Genome Res.

[76] Kong A, Thorleifsson G, Gudbjartsson DF, Masson G, Sigurdsson A, Jonasdottir A, Walters GB, Jonasdottir A, Gylfason A, Kristinsson KT, Gudjonsson SA, Frigge ML, Helgason A, Thorsteinsdottir U, Stefansson K (2010). Fine-scale recombination rate differences between sexes, populations and individuals. Nature.

[77] Hinch AG, Tandon A, Patterson N, Song Y, Rohland N, Palmer CD, Chen GK, Wang K, Buxbaum SG, Akylbekova EL, Aldrich MC, Ambrosone CB, Amos C, Bandera EV, Berndt SI, Bernstein L, Blot WJ, Bock CH, Boerwinkle E, Cai Q, Caporaso N, Casey G, Adrienne Cupples L, Deming SL, Ryan Diver W, Divers J, Fornage M, Gillanders EM, Glessner J, Harris CC (2011). The landscape of recombination in African Americans. Nature.

[78] Laayouni H, Montanucci L, Sikora M, Melé M, Dall’Olio GM, Lorente-Galdos B, McGee KM, Graffelman J, Awadalla P, Bosch E, Comas D, Navarro A, Calafell F, Casals F, Bertranpetit J (2011). Similarity in recombination rate estimates highly correlates with genetic differentiation in humans. PLoS One.

[79] Adzhubei I, Schmidt S, Peshkin L, Ramensky V, Gerasimova A, Bork P, Kondrashov A, Sunyaev S (2010). A method and server for predicting damaging missense mutations. Nat Methods.

[80] Ng PC, Henikoff S (2001). Predicting deleterious amino acid substitutions. Genome Res.

[81] Schwarz JM, Rodelsperger C, Schuelke M, Seelow D (2010). MutationTaster evaluates disease-causing potential of sequence alterations. Nat Meth.

[82] Chun S, Fay JC (2009). Identification of deleterious mutations within three human genomes. Genome Res.

[83] Boyle AP, Hong EL, Hariharan M, Cheng Y, Schaub MA, Kasowski M, Karczewski KJ, Park J, Hitz BC, Weng S, Cherry JM, Snyder M (2012). Annotation of functional variation in personal genomes using RegulomeDB. Genome Res.

[84] Ingenuity Pathway analysis: **Ingenuity® systems.** http://www.ingenuity.com

[85] Huang DW, Sherman BT, Lempicki RA (2009). Bioinformatics enrichment tools: paths toward the comprehensive functional analysis of large gene lists. Nucleic Acids Res.

[86] Amato R, Pinelli M, Monticelli A, Marino D, Miele G, Cocozza S (2009). Genome-wide scan for signatures of human population differentiation and their relationship with natural selection, functional pathways and diseases. PLoS One.

[87] Vernot B, Stergachis AB, Maurano MT, Vierstra J, Neph S, Thurman RE, Stamatoyannopoulos JA, Akey JM (2012). Personal and population genomics of human regulatory variation. Genome Res.

[88] Tennessen JA, Akey JM (2011). Parallel adaptive divergence among geographically diverse human populations. PLoS Genet.

[89] Biankin AV, Waddell N, Kassahn KS, Gingras M-C, Muthuswamy LB, Johns AL, Miller DK, Wilson PJ, Patch A-M, Wu J, Chang DK, Cowley MJ, Gardiner BB, Song S, Harliwong I, Idrisoglu S, Nourse C, Nourbakhsh E, Manning S, Wani S, Gongora M, Pajic M, Scarlett CJ, Gill AJ, Pinho AV, Rooman I, Anderson M, Holmes O, Leonard C, Taylor D (2012). Pancreatic cancer genomes reveal aberrations in axon guidance pathway genes. Nature.

[90] Chédotal A, Kerjan G, Moreau-Fauvarque C (2005). The brain within the tumor: new roles for axon guidance molecules in cancers. Cell Death Differ.

[91] Mehlen P, Delloye-Bourgeois C, Chédotal A (2011). Novel roles for slits and netrins: axon guidance cues as anticancer targets?. Nat Rev Cancer.

[92] **1000 Genome Project FTP server** ftp://ftp.1000genomes.ebi.ac.uk/vol1/ftp/phase1/analysis_results/integrated_call_sets/

[93] Danecek P, Auton A, Abecasis G, Albers CA, Banks E, DePristo MA, Handsaker RE, Lunter G, Marth GT, Sherry ST, McVean G, Durbin R, Genomes Project Analysis G (2011). The variant call format and VCFtools. Bioinformatics.

[94] Purcell S, Neale B, Todd-Brown K, Thomas L, Ferreira MAR, Bender D, Maller J, Sklar P, de Bakker PIW, Daly MJ, Sham PC (2007). PLINK: a tool Set for whole-genome association and population-based linkage analyses. Am J Hum Genet.

[95] **WHAMM** http://coruscant.itmat.upenn.edu/whamm/index.html

[96] Matise TC, Chen F, Chen W, De La Vega FM, Hansen M, He C, Hyland FCL, Kennedy GC, Kong X, Murray SS, Ziegle JS, Stewart WCL, Buyske S (2007). A second-generation combined linkage–physical map of the human genome. Genome Res.

[97] Kent WJ, Sugnet CW, Furey TS, Roskin KM, Pringle TH, Zahler AM, Haussler D (2002). The human genome browser at UCSC. Genome Res.

[98] Wang K, Li M, Hakonarson H (2010). ANNOVAR: functional annotation of genetic variants from high-throughput sequencing data. Nucleic Acids Res.

[99] Sherry ST, Ward M-H, Kholodov M, Baker J, Phan L, Smigielski EM, Sirotkin K (2001). dbSNP: the NCBI database of genetic variation. Nucleic Acids Res.

[100] **Online Mendelian Inheritance in Man, OMIM®** http://omim.org/17642958

[101] Kanehisa M, Goto S (2000). KEGG: Kyoto encyclopedia of genes and genomes. Nucleic Acids Res.

[102] Kanehisa M, Goto S, Sato Y, Furumichi M, Tanabe M (2012). KEGG for integration and interpretation of large-scale molecular data sets. Nucleic Acids Res.

[103] **GeneCards database** http://www.genecards.org/

